# Early postoperative tumor progression predicts clinical outcome in glioblastoma—implication for clinical trials

**DOI:** 10.1007/s11060-016-2362-z

**Published:** 2017-01-18

**Authors:** Andreas Merkel, Dorothea Soeldner, Christina Wendl, Dilek Urkan, Joji B. Kuramatsu, Corinna Seliger, Martin Proescholdt, Ilker Y. Eyupoglu, Peter Hau, Martin Uhl

**Affiliations:** 1 Department of Neurosurgery, Friedrich-Alexander University Erlangen-Nuremberg, Erlangen, Germany; 2 Department of Neurology and Wilhelm Sander-NeuroOncology Unit, University of Regensburg, Regensburg, Germany; 3 Department of Neuroradiology, University of Regensburg, Regensburg, Germany; 4 Department of Neurology, University Hospital, Friedrich-Alexander University Erlangen-Nuremberg, Schwabachanlage 6, 91054 Erlangen, Germany; 5 Department of Neurosurgery, University of Regensburg, Regensburg, Germany

**Keywords:** Glioblastoma, MGMT promoter, Treatment delay, Magnetic resonance imaging, Survival

## Abstract

Molecular markers define the diagnosis of glioblastoma in the new WHO classification of 2016, challenging neuro-oncology centers to provide timely treatment initiation. The aim of this study was to determine whether a time delay to treatment initiation was accompanied by signs of early tumor progression in an MRI before the start of radiotherapy, and, if so, whether this influences the survival of glioblastoma patients. Images from 61 patients with early post-surgery MRI and a second MRI just before the start of radiotherapy were examined retrospectively for signs of early tumor progression. Survival information was analyzed using the Kaplan–Meier method, and a Cox multivariate analysis was performed to identify independent variables for survival prediction. 59 percent of patients showed signs of early tumor progression after a mean time of 24.1 days from the early post-surgery MRI to the start of radiotherapy. Compared to the group without signs of early tumor progression, which had a mean time of 23.3 days (p = 0.685, Student’s *t* test), progression free survival was reduced from 320 to 185 days (HR 2.3; CI 95% 1.3–4.0; p = 0.0042, log-rank test) and overall survival from 778 to 329 days (HR 2.9; CI 95% 1.6–5.1; p = 0.0005). A multivariate Cox regression analysis revealed that the Karnofsky performance score, O-6-methylguanine-DNA-methyltransferase (MGMT) promoter methylation, and signs of early tumor progression are prognostic markers of overall survival. Early tumor progression at the start of radiotherapy is associated with a worse prognosis for glioblastoma patients. A standardized baseline MRI might allow for better patient stratification.

**Electronic supplementary material:**

The online version of this article (doi:10.1007/s11060-016-2362-z) contains supplementary material, which is available to authorized users.

## Introduction

Malignant gliomas are the most common brain tumor entity, and from those, glioblastoma is one of the most threatening. The current first-line treatment protocol includes surgery followed by combined radio- and chemotherapy [1]. Although this treatment protocol is very aggressive, the median survival time of 14 months reflects a poor prognosis. It is a common sentiment that an early treatment initiation is important for optimal tumor control. Nevertheless, reliable prospective data supporting this are lacking. Where one retrospective analysis indicated that delaying radiotherapy increased the risk of death by 9% weekly [2], others showed no evidence of an effect on overall survival [3, 4]. Today most oncologists would agree that delaying the initiation of radiotherapy for up to 4 weeks after tumor resection is not harmful [5, 6].

Given the dismal prognosis, new therapeutic concepts are needed urgently and require prospective evaluation within clinical trials. In the neuro-oncological field, reference histology and molecular marker evaluation with O-6-methylguanine-DNA-methyltransferase (MGMT) promoter methylation are now widely used for molecular analysis of glioblastoma for inclusion in clinical trials (e.g. Centric, Glarius). It is likely that up-and-coming markers such as the mutated isocitrate dehydrogenase, which is now an integral standard in the amended WHO classification [7], will also require more upfront testing time for patients in the future. This implies a potential critical delay in treatment initiation that could endanger the treatment outcome, especially if the markers are evaluated centrally within a clinical trial [8].

We examined retrospectively the MRIs and clinical course of 61 glioblastoma patients in their first-line treatment and addressed whether the MRI signs of early tumor progression, which occur during the waiting time to treatment initiation, are prognostic of survival.

## Materials and methods

### Patient selection

We screened our database from the years 2009 to 2013 for patients with a histologically confirmed diagnosis of glioblastoma that had post-surgery MRI as well as a baseline MRI before start of radiotherapy. Patients were identified according to the inclusion criteria of having a well-documented clinical course and sufficient data for analysis, a post-surgery MRI, and a baseline MRI. As a substantial number of patients (22) was registered within clinical trials, we excluded patients >75 years, with Karnofsky performance status <70, or with surgical complications that would have interfered with a participation in a clinical trial, leaving a total of 61 patients for analysis.

### Treatment regimens

Patients treated within clinical trials gave informed consent and were treated according to the treatment plans. At the date of writing this manuscript, two of the three clinical trials are already published as negative trials with no effect on overall survival, leaving a total of three patients that received an experimental chemotherapy of unpublished activity. Patients within the clinical routine also gave informed consent for further scientific analysis of their clinical dataset and were treated according to the local guidelines with combined radio- and chemotherapy with Temozolomide [1]. The study design was approved by the local ethics committee under the registration number 14-101-0035.

### Imaging procedures

Initial diagnostic, intra-surgery or post-surgery, baseline and follow-up MRIs were used for analysis according to hospital guidelines. In brief, T1 contrast-enhanced sequences were used to localize the tumor and possible early recurrence by an experienced radiological specialist (CW) and a neuro-oncologist (MU). New T1 contrast-enhancing lesions distant from the resection cavity, new nodular contrast-enhancing lesions at the border of the resection cavity and an increase in residual tumor were considered signs of early tumor progression.

### Tumor progression and overall survival

Progression-free survival was defined as the time from surgery to the first tumor recurrence after surgery. It was censored when death occurred before MRI detected progression or if lost to follow-up. Overall survival was defined as the time from initial surgery to death. If death did not occur at the time of data lockup in April 2016, events were marked as censored.

### Statistical analysis

Gender, age, Karnofsky performance score (KPS), extent of resection, MGMT promoter methylation, waiting time and first-line and second-line therapy were registered retrospectively using the original patient charts and trial documentation. Differences between the patient group for early tumor progression and the group with stable disease at baseline MRI were analyzed using the Fisher’s exact test for frequency distributions, and, after testing for normal distribution, the *t* test or the Mann-Whitney-U-test, as appropriate. Progression-free and overall survival was analyzed using the Kaplan–Meier method. Differences between the two groups were analyzed for statistical significance by using the log-rank test. Age, KPS, extent of resection, MGMT promoter methylation and signs of early tumor progression were included in a multivariate Cox regression analysis for overall survival. Correlation between delay to baseline MRI and overall survival was analyzed using the Spearman’s ρ method. Receiver operating characteristic (ROC) analysis with Youden’s J statistic was used to discern the best cut-off time to detect signs of early tumor progression [9]. All statistical analyses were performed using SPSS and Graph Pad (JBK and IYE).

## Results

### Signs of early tumor progression at baseline MRI

We analyzed our dataset of 61 patients with a 24 h post-surgery and a baseline MRI just before initiation of radiotherapy for signs of early tumor progression. 36 of 61 patients (59%) showed signs of early tumor progression. 9 of 36 patients (25%) had a distant new lesion not directly associated with the original resection site, 27 of 36 patients (75%) showed a new lesion in the vicinity of the resection cavity, and 28 of 36 patients (78%) had a progression of residual tumor (see Supplementary Fig. 1 for illustration). The mean waiting time in the group with signs of early tumor progression to baseline MRI was 24.1 days; in the group with no progress it was 23.3 days (p = 0.685; Student’s *t* test).

### Patient characteristics

Before addressing a possible effect on survival we characterized both groups with and without signs of early tumor progression for confounding variables. The groups were evenly distributed for gender, age, Karnofsky performance scale (KPS), MGMT promoter methylation, delay to initiation of radiotherapy, participation in clinical trials and bevacizumab use as outlined in Table 1. Concerning the extent of resection the group with early tumor progression had more biopsies (n = 12/36 (33%) vs. n = 1/25 (4%); p = 0.009; Fisher’s exact test) and had fewer patients receiving adjuvant chemotherapy (n = 24/36 (67%) vs. n = 23/25 (92%); p = 0.030; Fisher’s exact test) and second-line surgery (n = 5/35 (14%) vs. n = 11/25 (44%); p = 0.016; Fisher’s exact test).

**Table 1:** Patient characteristics of the two groups with and without signs of early tumor progression

Characteristics	Early tumor progression n = 36	No progression n = 25	Significance
Gender			
Female	19 (53%)	10 (40%)	p = 0.435
Age in years (SD)	56.3 (11, 4)	58.2 (9, 3)	p = 0.503
KPS (IQR)	80 (80–90)	80 (80–90)	p = 0.671
Extent of resection			
Total	2 (6%)	4 (16%)	p = 0.216
Partial	22 (61%)	20 (80%)	p = 0.163
Biopsy only	12 (33%)	1 (4%)	p = 0.009
MGMT promotor status			
Available	29 (81%)	19 (76%)	
Methylated	10 (35%)	4 (21%)	p = 0.354
Waiting time to			
Baseline MRI in days (SD)	24.1 (7, 1)	23.3 (6, 6)	p = 0.685
Radiotherapy (SD)	29.9 (7, 5)	30.9 (6, 1)	p = 0.607
Patients treated within experimental protocols	15 (42%)	7 (28%)	p = 0.170
First line therapy			
Adj. chemotherapy	24 (67%)	23 (92%)	p = 0.030
Numbers of TMZ cycles (IQR)	4 (2–6)	6 (4–8)	p = 0.143
Bevacizumab use at any time	12 (33%)	7 (28%)	p = 0.781
Second line therapy			
2nd surgery	5 (14%)	11 (44%)	p = 0.016
2nd radiotherapy	8 (22%)	8 (32%)	p = 0.555
2nd line chemotherapy	16 (44%)	14 (56%)	p = 0.440

*KPS* Karnofsky performance status, *MGMT* O6-methylguanine-DNA methyltransferase, *adj*. adjuvant, *TMZ* temozolomide, *IQR* interquartile range, *SD* standard deviation

Statistical analysis: age, extent of resection, experimental protocols, adj. chemotherapy, bevacizumab use, and second line therapy, Fisher’s exact test; age, and waiting time, *t* test; gender, and numbers of TMZ cycles, Man Whitney U test

### Influence of early tumor progression on survival

**Fig. 1:**
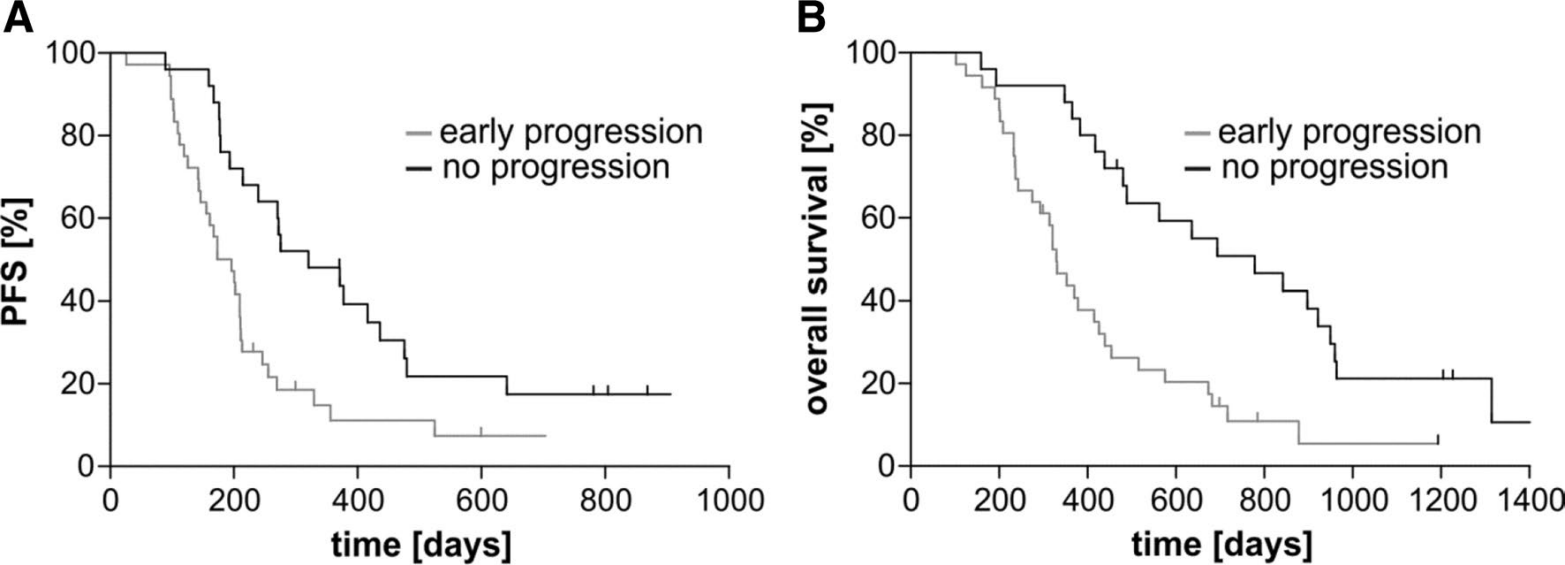
Influence of early tumor progression on survival. Panel **a** shows progression-free survival in days from surgery to first progression, and Panel **b** shows overall survival for patients showing signs of early tumor progression (early progression) or not (no progression) at baseline MRI. In **a**, the median was 185 and 320 days, HR 2.3; CI 95% [1.3–4.0]; p = 0.0042, and in **b**, the median was 329 and 776 days, HR 2.9; CI 95% [1.6–5.1]; p = 0.0005, log-rank test

We next asked the question whether the 36 of 61 patients that already showed a tumor recurrence at baseline MRI had a worse prognosis or not. The group with no signs of early tumor progression showed a median progression-free survival (PFS) of 320 days (Fig. 1a). Patients with early tumor progression had a median PFS of 185 days, translating into a hazard ratio of 2.3 (CI 95% 1.3–4.0; p = 0.0042; log-rank test). Similar results were found for overall survival (OS) in Fig. 1b. Patients with no signs of early tumor progression had a median OS of 778 days, whereas patients with early progression had a median of 329 days, with a hazard ratio of 2.9 (CI 95% 1.6–5.1; p = 0.0005; log-rank test). With respect to the localization of the early tumor progression, there was no difference in terms of PFS or OS for an early tumor progression in the vicinity of or distant from the resection cavity (data not shown).

### Early tumor progression is an independent prognostic marker for overall survival

As the groups with and without signs of early tumor progression had an imbalance for prognostic markers like extent of resection [10] and adjuvant chemotherapy [1], we asked whether signs of early tumor progression is still an independent marker for survival outcome. We therefore conducted a multivariate Cox regression analysis with age, KPS, extent of resection, MGMT promoter methylation and signs for early tumor progression as independent variables (Table 2). KPS (p = 0.005), MGMT promoter methylation (p < 0.0001) and early tumor progression (p = 0.001) were revealed to be significant markers. Age that had been limited by the inclusion criteria to <75 years was not significant (p = 0.182). Extent of resection defined by biopsy also showed no significance (p = 0.484) in our data set.

**Table 2:** Multivariate Cox regression analysis for overall survival

Covariable	OR	95% CI	Significance
Age	0.962	0.908–1.018	p = 0.182
KPS	1.090	1.027–1.196	p = 0.005
Extent of resection (not biopsy)	1.512	0.476–4.804	p = 0.484
MGMT	16.946	3.687–77.898	p < 0.0001
Early tumor progression	0.182	0.066–0.502	p = 0.001

*KPS* Karnofsky performance status, *MGMT* O6-methylguanine-DNA methyltransferase, *OR* odds ratio, *CI* confidence interval

### For patients with a documented early tumor progression at baseline MRI, the time delay to the initiation of radiotherapy correlates with overall survival

**Fig. 2:**
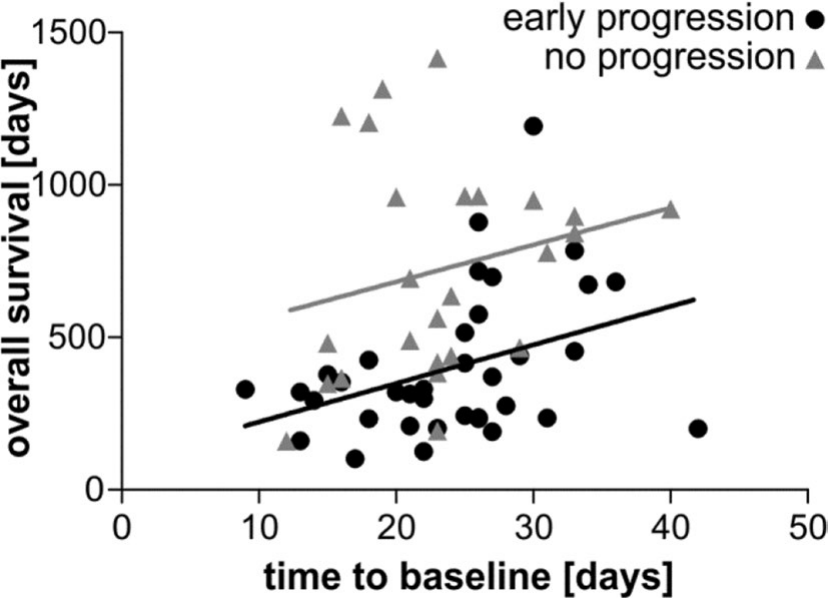
Correlation between delay to baseline MRI and OS. The correlation between the waiting time to baseline MRI and OS is shown. Only the group with signs of early tumor progression showed a significant correlation between the time delay to baseline MRI and overall survival, with patients showing signs of early tumor progression at earlier time points having a worse prognosis. Spearman’s ρ test; p = 0.023

Given the assumption of a linear tumor growth and a stable detection limit defined by MRI technology, there should be a theoretical time point when tumor growth can be first detected. We therefore asked whether the presence of signs of tumor progression at an early baseline MRI compared to a late time point would be reflective of the tumor growth velocity and ultimately its prognosis. There was a significant correlation between the time to baseline MRI and OS in the group with signs of early tumor progression (Fig. 2; p = 0.023 Spearman’s ρ), whereas for the patients without progression these factors did not correlate. This suggests that patients with detected tumor growth in two consecutive MRIs in a short time window have a worse prognosis compared to patients for which the time window is extended. In contrast, if no early tumor progression was detected at baseline MRI, timing was irrelevant for prognosis.

### Optimal time point for baseline MRI

Current clinical trials in neuro-oncology demand only in the cases of treatment delayed beyond 28 days an additional baseline MRI following the post-surgery MRI. Knowing the correlation between the time delay to baseline MRI and OS, we asked if there is an ideal time point to check for signs of early tumor progression. We therefore performed a ROC analysis, which was not significant. Cautiously interpreted, one might consider the Youden’s index of 0.236 as an indicator that 24 days are a cutoff point to determine signs of early tumor progression.

## Discussion

The data presented analyzed the relevance of early tumor progression and contributed to the long debate on the ideal timing of adjuvant therapy in the treatment of glioblastoma patients. As outlined in the introduction, there are conflicting reports in the literature. The latest report by Han et al. advocates a time window for treatment initiation [5]. Surprisingly studies examining the prognostic value of time delay to treatment did not use imaging to search for signs of tumor progression.

Although this study is a retrospective analysis, to our knowledge it is the first report examining consecutive MRIs for prognostic signs before the initiation of radiotherapy. There is a report of Stensjoen et al. that examined the growth dynamics of untreated glioblastomas before initial surgery [11]. This group reported a growth rate of 1.4% per day and a volume doubling time of 50 days. They concluded that poor treatment logistics influence tumor size before surgery. Pennington et al. examined MRIs between surgery and the initiation of radiotherapy, similar to our approach [12]. They observed a median time delay of 31.5 days between the scans and calculated a growth of 35%, comparable to the report by Stensjoen et al. The conclusion was that given the growth kinetics, it is unlikely that tumor cells outgrew the usual 2–3 cm margin for radiotherapy within the given timeframe. Both groups did not correlate growth kinetics with clinical outcome parameters like PFS and OS.

A correlation done by Gladwish et al. comparing post-surgery and post-radiotherapy MRIs allowed a prognostic prediction, similar to our results [13]. The timing of the analyzed consecutive MRIs over radiotherapy raises the question of pseudo-progression or pseudo-responses and possibly clouds the predictive value of the MRIs. Adding advanced MRI techniques including MRI perfusion and cerebral blood volume [14] or MRI spectroscopy [15] did not completely help in discriminating true progress from pseudo-progress in this time setting before and after radiotherapy.

An advantage of our study is that timing two MRIs before the initiation of radiotherapy excludes any therapeutic influence. We show that at the time radiotherapy is initiated at least 60% of patients already show signs of tumor recurrence, which comes with a poor prognosis, raising the question of whether to repeat surgery [16]. On the other hand, one can argue that early surgical re-resection will not correct the prognosis, and that early detection of tumor recurrence reflects the more aggressive nature of some glioblastomas. Unfortunately the 61 patients in our trial were not a sufficiently large cohort for an ROC analysis determining an ideal time point to discriminate between the presence and absence of early tumor progression. More patients, ideally in a prospective trial with advanced MRI techniques or FET-PET, could address this.

Finally we have to raise questions concerning how clinical studies into glioblastoma are currently conducted. Most protocols require an early (ideally within 24 h) post-surgery MRI and, only if a time delay exceeds 28 days to radiotherapy, a baseline MRI (e.g. Centric, Director, Glarius). Given the additional information from our study we believe that all patients should have a baseline MRI as an independent prognostic marker for stratification. An additional theme to mention here is the time point of randomization. Some trials randomize early at the initiation of radiotherapy (e.g. Centric, Glarius, Checkmate), while others randomize after the completion of radiotherapy (e.g. ACT IV, Novocure). Not surprisingly, these trials are hard to compare as the latter exclude patients that already show tumor recurrence. A mandatory baseline MRI, at a still-to-be-determined fixed time point before radiotherapy, would eliminate this and allow for comparison between early and late randomization trials.

In summary we show that 60% of glioblastoma patients in their first-line therapy experienced tumor progression as early as at the initiation of their radiotherapy. This recurrence is associated with a worse prognosis. We advocate for a standard baseline MRI to detect this unfavorable early course of disease both within and outside of clinical trials. The ideal time point of this MRI must be determined in further studies.

## Electronic supplementary material

Below is the link to the electronic supplementary material.

Supplementary Fig. 1 Examples of T1 contrast enhanced early post-surgery and baseline MRIs. Panel A shows an example of no signs of early tumor progression at the resection site. In Panel B, an arrow marks small and nodular contrast enhancement distant from the resection cavity. In Panel C, residual tumor progress is marked with an asterisk (*). The double asterisks (**) in C mark a new nodular contrast enhancement. Panel D shows multiple combinations of the above-mentioned patterns of recurrence at initiation of radiotherapy. (PDF 227 KB)
