# Supplementary figures and images for: Early postoperative tumor progression predicts clinical outcome in glioblastoma—implication for clinical trials
Source: J Neurooncol. 2017 Jan 18;132(2):249–54. doi: 10.1007/s11060-016-2362-z (PMC5378726; doi:10.1007/s11060-016-2362-z)

post-surgery

baseline

post-surgery

baseline

**A**

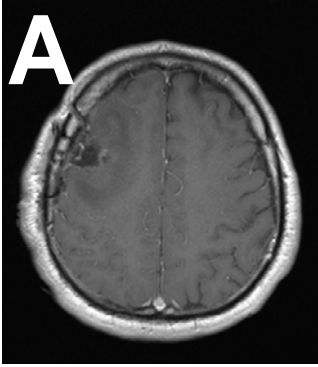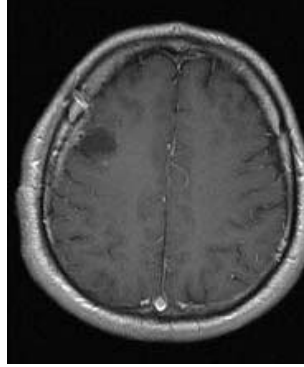

**B**

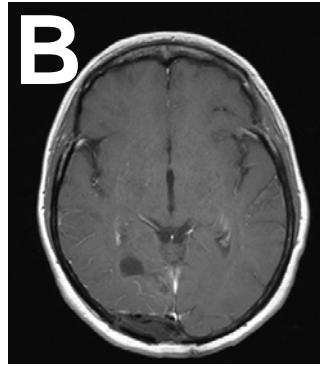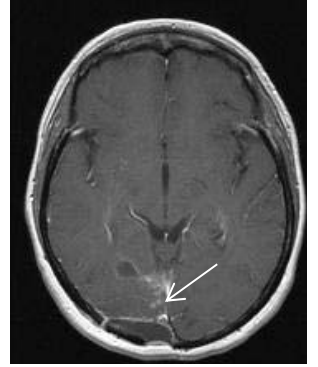

**C**

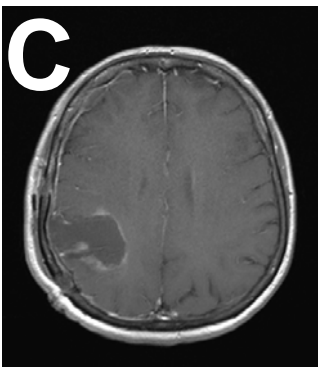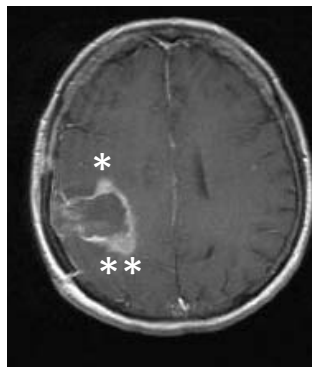

**D**

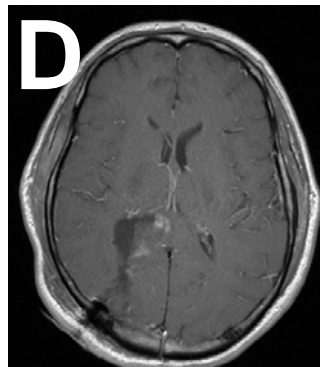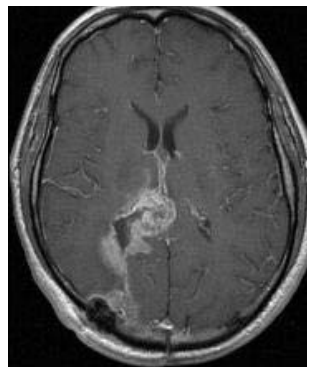

Supplement: Supplementary file 1 — Supplementary Fig. 1 Examples of T1 contrast enhanced early post-surgery and baseline MRIs. Panel A shows an example of no signs of early tumor progression at the resection site. In Panel B, an arrow marks small and nodular contrast enhancement distant from the resection cavity. In Panel C, residual tumor progress is marked with an asterisk (*). The double asterisks (**) in C mark a new nodular contrast enhancement. Panel D shows multiple combinations of the above-mentioned patterns of recurrence at initiation of radiotherapy. (PDF 227 KB) [file 11060_2016_2362_MOESM1_ESM.pdf]
